# Unraveling the complexities of educational and socioeconomic factors in cognitive decline with an AI model

**DOI:** 10.1002/alz.095318

**Published:** 2025-01-09

**Authors:** Paola Ruiz Puentes, Nestor González, Daniel Crovo, Beatriz Grimaldi, Ram Mukunda

**Affiliations:** ^1^ IGC Pharma, Bogotá, Bogotá D.C Colombia; ^2^ IGC Pharma, Potomac, MD USA

## Abstract

**Background:**

High formal education level is widely recognized as a protective factor against cognitive decline, influenced by the reserve hypothesis. However, in multiple studies educational experience is confounded with cultural and socioeconomic factors, leading to contradictory findings. Factors such as an individual’s living environment, including socioeconomic conditions, rather than individual variables such as education, are hypothesized to be factors influencing cognitive decline. This study leverages data from the Mexican Health and Aging Study (MHAS) and the Mexican Cognitive Aging Ancillary Study (Mex‐Cog), which provide information on the living conditions of older adults and their cognitive status. Through artificial intelligence models, this research aims to provide a nuanced understanding of cognitive decline risk factors as it relates to educational and socioeconomic factors.

**Methods:**

The Mexican Health and Aging Study (MHAS) provides national‐level data on individuals aged 50 and over across six waves from 2001 to 2021, including detailed cognitive assessments (Mex‐Cog) in 2016 and 2021. We trained a traditional machine learning and a deep learning algorithm, specifically a random forest and a transformer‐based model, to predict participants' Mini‐Mental State Examination (MMSE) scores. For this task we used a comprehensive dataset that includes historical and current data on demographics, health, health care utilization and insurance, cognition, financial and housing wealth, income, pension, family structure, employment, assistance and caregiving, stress and psychosocial scores, and end of life planning. We employed Shapley‐based analyses to identify the most influential factors and their interactions in the MMSE score predictions. We present our initial findings on factors influencing cognitive decline.

**Results:**

Certain algorithms, especially random forests are superior to other algorithms that we tried to use. Our approach, while limited to the Mexican data, shows, for example, education plus income as a superior indicator of risk for cognitive decline than education alone.

**Conclusion:**

Our model, with further analysis, has far‐reaching public policy implications. We continue to expand the algorithm to include multiple factors beyond education to assess risk of cognitive decline.

